# Hysteresis Compensation of Piezoresistive Carbon Nanotube/Polydimethylsiloxane Composite-Based Force Sensors

**DOI:** 10.3390/s17020229

**Published:** 2017-01-24

**Authors:** Ji-Sik Kim, Gi-Woo Kim

**Affiliations:** 1 School of Nano & Advanced Material Engineering, Kyungpook National University, Sang-Ju, 37224, Korea; jisikkim@knu.ac.kr; 2 Department of Mechanical Engineering, Inha University, Incheon 22212, Korea

**Keywords:** carbon nanotube, polydimethylsiloxane, nanocomposite, piezoresistive effect, hysteresis compensation, force sensor

## Abstract

This paper provides a preliminary study on the hysteresis compensation of a piezoresistive silicon-based polymer composite, poly(dimethylsiloxane) dispersed with carbon nanotubes (CNTs), to demonstrate its feasibility as a conductive composite (i.e., a force-sensitive resistor) for force sensors. In this study, the potential use of the nanotube/polydimethylsiloxane (CNT/PDMS) as a force sensor is evaluated for the first time. The experimental results show that the electrical resistance of the CNT/PDMS composite changes in response to sinusoidal loading and static compressive load. The compensated output based on the Duhem hysteresis model shows a linear relationship. This simple hysteresis model can compensate for the nonlinear frequency-dependent hysteresis phenomenon when a dynamic sinusoidal force input is applied.

## 1. Introduction

Over the last few decades, carbon nanotubes (CNTs) have attracted significant attention because their excellent mechanical properties, which can be applied for various sensor engineering applications [1]. In particular, CNTs enable us to find potential sensor applications when used as additives for various matrix materials because of their high thermal conductivity and attractive electrical properties. For instance, CNTs can create a conductive network at concentrations as low as 0.1 wt % because of their high aspect ratio (>500) [2]. CNT-filled composites typically exhibit interesting electromechanical properties. For example, the electrical resistivity can change in response to different mechanical stimuli (e.g., stress), which is commonly called the piezoresistive effect. This effect has primarily been studied in composite materials consisting of conductive fillers and a polymer matrix. Carbon black nanocomposites have also been studied as potential conductive fillers for tensile strain and pressure-sensor materials [3].

CNTs also can be dispersed into cements with an aqueous solvent, and their piezoresistive properties have been experimentally evaluated at a high stress level of 6 MPa for traffic flow monitoring [4]. Cho et al. [5] reported an experimental study on the piezoresistive effect of a synthetic rubber such as styrene–butadiene rubber (SBR)-based composite mixed with multi-walled CNTs to examine its feasibility as an embedded force sensor for automotive smart tires.

Meanwhile, flexible CNT/polymer composites have been studied for many potential applications, such as haptic skins, wearable electronics, and stress/strain sensors for structural health monitoring [6,7,8,9]. Polydimethylsiloxane (PDMS) silicone has gained significant interest as one of the promising CNT composites because of its excellent flexibility, making it suitable for MEMS technology. For example, the conductive multiwalled carbon nanotubes (MWCNTs) dispersed into a PDMS matrix was studied in detail by Lu et al. [10]. The mechanical and electromechanical properties of this material under different conditions have been characterized. The electrical resistance-pressure sensitivity of MWCNT networks/PDMS composites have also been explored [11]. Recently, a stretchable and transparent strain sensor made of an ultrathin film of single-walled carbon nanotubes (SWNTs) encapsulated inside PDMS films was proposed [12]. However, there have been no studies on hysteresis and its compensation for CNT/polymer composites including CNT/PDMS despite hysteresis being a commonly occurring phenomenon in sensor applications. Moreover, the majority of the studies on CNT/polymer composites focus on sensor applications at low stress (force) levels.

The primary objective of this research is, therefore, to study the hysteresis compensation of a piezoresistive silicon-based polymer composite, PDMS dispersed within carbon nanotubes (CNTs), and to demonstrate its feasibility as a conductive composite (i.e., a force-sensitive resistor) for high capacity force sensors. In Section 2, a brief fabrication method and fundamental properties of CNT/PDMS composite are also presented. The CNT/PDMS composite was characterized by measuring the piezoresistive response to a sinusoidal loading as well as a static compressive load, as described in Section 3. Finally, as discussed in Section 4, the Duhem hysteresis model was proposed to compensate for the nonlinear frequency-dependent hysteresis phenomenon, and its effectiveness was experimentally validated.

## 2. Fabrication of the CNT/PDMS Composite

**Figure 1:**
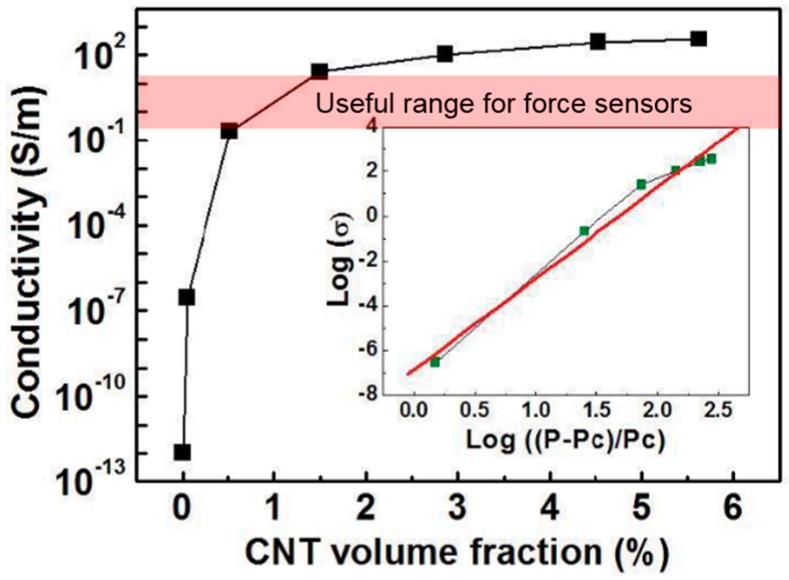
The electrical conductivity of the CNT/PDMS composites as a function of CNT concentration (adapted from Ref. [14]).

**Figure 2:**
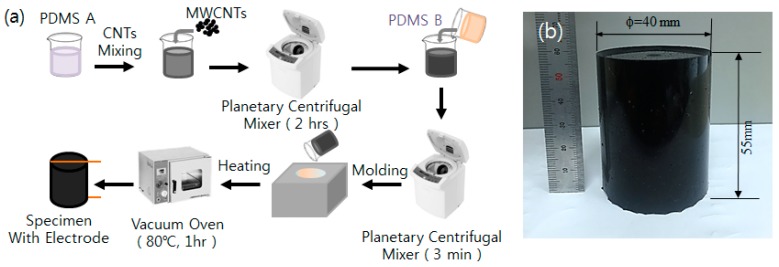
CNT/PDMS composite sample: (**a**) fabrication process and (**b**) photograph.

To fabricate CNT/PDMS composites, PDMS (Dow Corning^®^ SYLGARD 184) was mixed with high-purity (99 wt %) multi-walled carbon nanotubes (MWCNTs) manufactured by Carbon Nano-material Technology Co., Ltd (model: MR99) [13]. For the conductive CNT/PDMS composite samples, the measured conductivity (i.e., the inverse of electrical resistance) highly depends on the CNT concentration. MWCNTs, with an average diameter of 10 nm, should be fully dispersed within the matrix (e.g., PDMS) to build a conductive network and utilize their electrical properties. The available concentration range of CNT can be determined from the conductivity of CNT/PDMS composite as a function of weight volume of MWCNTs, as shown in Figure 1. Note that the percolation threshold of CNT/PDMS composite is approximately 0.1% in Figure 1. If the concentration of CNT is around the percolation threshold, the resistance must be high and it becomes difficult to distinguish the signal from the electrical noises. However, the sensor output will be impeded by the contact resistance if the conductivity is too high (i.e., resistance is too low). Therefore, the CNT concentration was selected to be approximately 1 wt % from the useful range of conductivity, as indicated in Figure 1. A high-energy, planetary centrifugal mixer (model: BSG-D1000RS, BYTECH) was used to homogeneously disperse CNTs within the PDMS. In addition, the constant local resistance was also verified by using partially fragmented specimens during the experiment. The MWCNTs and PDMS-A were then simply mixed using the planetary centrifugal mixer for 2 h and cured by a cross-linking agent with PDMS-B, as shown in Figure 2a. Then, the mixture was molded into a cylindrical ingot and dried in an oven at 80 °C for one hour. Figure 2b shows the dimensions of the fabricated sample of the CNT/PDMS composite.

**Figure 3:**
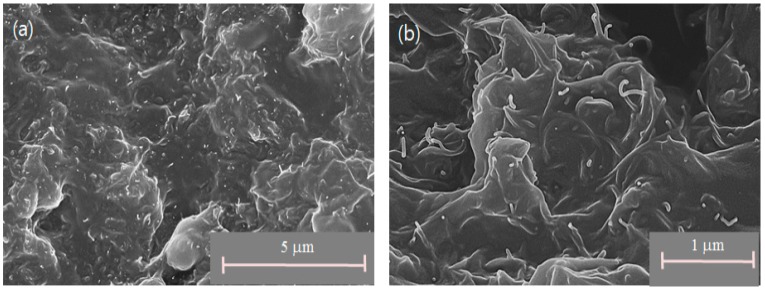
SEM images of the CNT/PDMS composite, with magnification: (**a**) 10,000× and (**b**) 30,000×.

**Figure 4:**
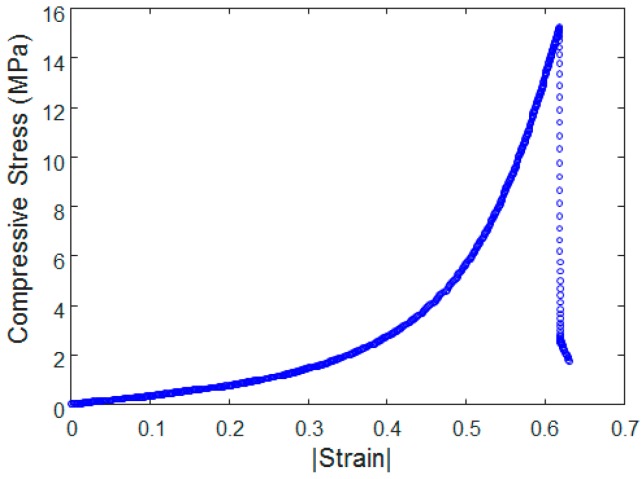
Strain versus compressive stress curves of the CNT/PDMS composite (1 wt %).

A scanning electron microscope (SEM, model: Hitachi S-4300SE, Tokyo, Japan) was utilized to closely examine the conductive networks of the CNT/PDMS composite. The SEM image in Figure 3 demonstrates that by simply mixing, CNTs can be uniformly dispersed into the PDMS matrix and can successfully form a network. In addition, the mechanical strength of the composite sample was evaluated with an electrodynamic testing instrument (model: Instron^®^ E3000, Norwood, MA, USA). Figure 4 shows the measured stress versus strain curves of the CNT/PDMS composite under uniaxial compressive loading. The compressive stress–strain curve of polymeric silicone typically exhibits an initial region of linear elasticity. Young’s modulus of such specimens was measured to be 6 MPa as the gradient of the linear portion of the stress–strain curve at a strain level of 0.3. As the strain further increases, the compressive stress rises quickly up to the maximum compressive stress, which is estimated to be 15 MPa at a strain of approximately 0.6.

## 3. Characterization: Piezoresistive Response

### 3.1. Experiment Set-Up

**Figure 5:**
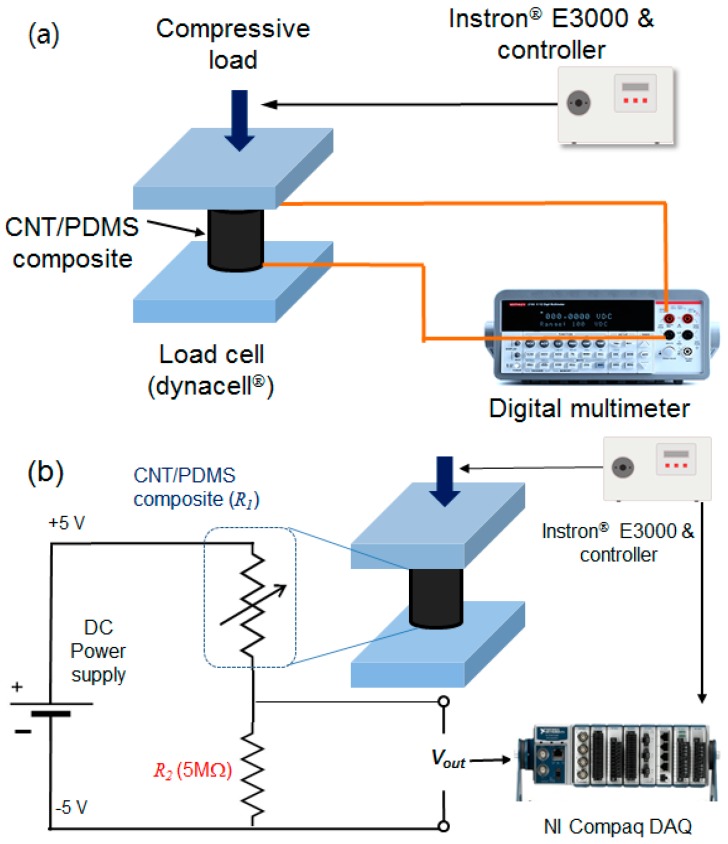
Experimental setup for measuring the (**a**) electrical resistance and (**b**) and voltage output using a voltage divider circuit.

The piezoresistive effect can typically be characterized by the gauge factor (GF), which is defined as the slope of the change in electrical resistance versus strain curve (i.e., sensitivity) defined by [15]

(1) $$GF=\frac{\Delta R}{\varepsilon R}$$

where Δ*R* is the change in the resistance, and *R* and ε are the initial values of the resistance and strain, respectively. In this study, the relation between compressive force and electrical resistance using an electric circuit was used as an alternative way to characterize the piezoresistive behavior of the CNT/PDMS composite-based force sensor [16]. The electrodynamic test instrument, consisting of a load cell and advanced digital control electronics, was used to measure the electrical resistance change in response to compressive loading (force), as shown in Figure 5a. Because the maximum dynamic and static load capacities of the test instrument are ±3 kN and ±2 kN, respectively, the test instrument could apply a sufficient load to the CNT/PDMS composite. For the static test, the compressive load was applied to the composite specimen with an increment of 200 N at each load step; meanwhile, the electrical resistance was measured with a two-lead method using a digital multimeter (model: Keithley 2701, 6 1/2-digit).

**Figure 6:**
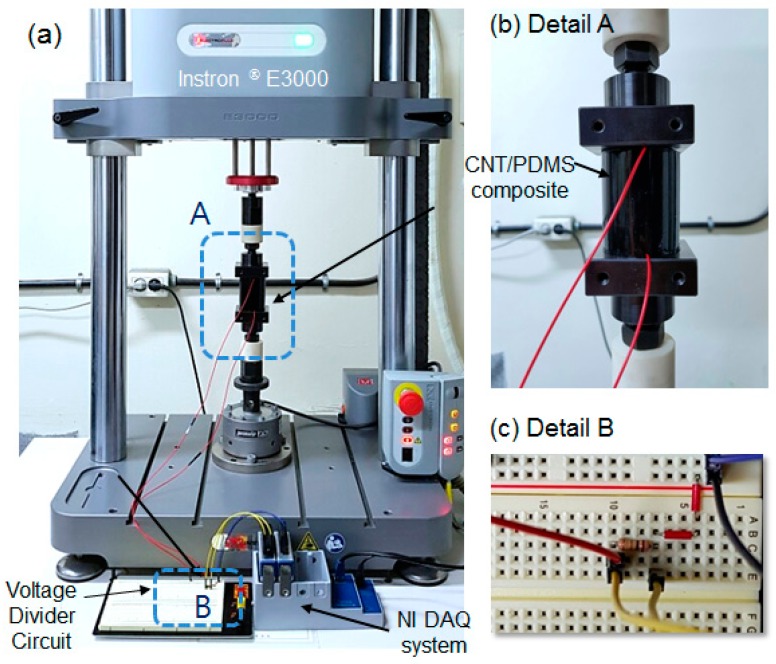
Photographs of the experimental setup: (**a**) overall configuration; (**b**) CNT/PDMS composite sample, and (**c**) voltage divider circuit.

Because the variable piezoresistive resistance ($R_{1}$) is inversely proportional to the applied static compressive load, a voltage divider circuit connecting a tuning resistor (*R_2_*, 5 MΩ in this study) and $R_{1}$ in series was used to convert the signal into a linear output, as shown in Figure 5b. This allows the CNT/PDMS composite to measure a variable resistance approximately proportional to external force, thus acting as a force sensor.

(2) $$V_{out}=\frac{R_{2}}{R_{1}+R_{2}}V_{in}$$

where $V_{in}$ is the supply DC voltage (5 V). The overall experimental setup includes the CNT/PDMS composite sample, electrodynamic test instrument, voltage divider circuit, and a data acquisition (DAQ) system (model: NI Compaq DAQ), as shown in Figure 6.

### 3.2. Results and Discussion

**Figure 7:**
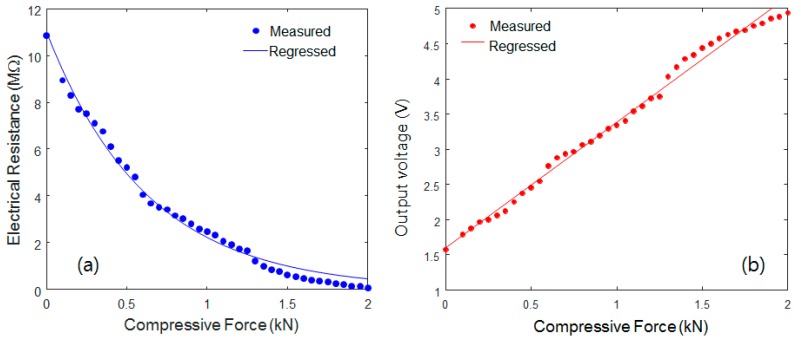
Static piezoresistive response to a step input. (**a**) Electrical resistance vs. compressive force and (**b**) output voltage vs. compressive force.

The electrical resistance was measured by the digital multimeter, as shown in Figure 7a. Because their variable piezoresistive resistance ($R_{1}$) was observed to be inversely proportional to the applied static compressive load, the CNT/PDMS composite exhibits a piezoresistive response. The relationship between the electrical resistance (*y-axis*) and input compressive force (*x-axis*) can be regressed by the following power function (square of the correlation coefficient *R*^2^ = 0.9899):

(3) $$y=ae^{bx}$$

where *a* is the coefficient (11.0), and *b* is the power exponent (−1.6). It has been reported that the piezoresistive mechanisms in various CNT strain sensors are mainly attributed to: (i) the tunneling effect due to the distance change between neighboring CNTs, (ii) the loss of contact among CNTs, and/or (iii) the deformation of CNTs [17,18]. The first tunneling resistance between two neighboring CNTs varies exponentially with the inter-distance between them. However, the variations of resistance are commonly linear in the second and third mechanisms. Therefore, it is believed (Figure 7) that the second and third mechanisms are not dominant, since the small strain of 1.0% applied to the specimen was not large enough to involve the deformation or loss of contact of the CNTs [18]. Consequently, the non-linear relationship between the input force and the electrical resistance might have been caused by the tunneling effects among the CNTs. While using the voltage divider circuit, the resistances expressed by Equation (3) were converted into a nearly linear relationship (i.e., compressive force versus output voltage), as shown in Figure 7b. The relationship between the output voltage (*y-axis*) and the input load (force) (*x-axis*) can also be regressed by the following function (square of correlation coefficient *R*^2^ = 0.9915).

(4) y=ax+b

where *a* is the gain (1.778) and *b* is the coefficient (1.608). Equation (4) can be used to obtain the sensitivity curve of the CNT/PDMS composite-based force sensors.

**Figure 8:**
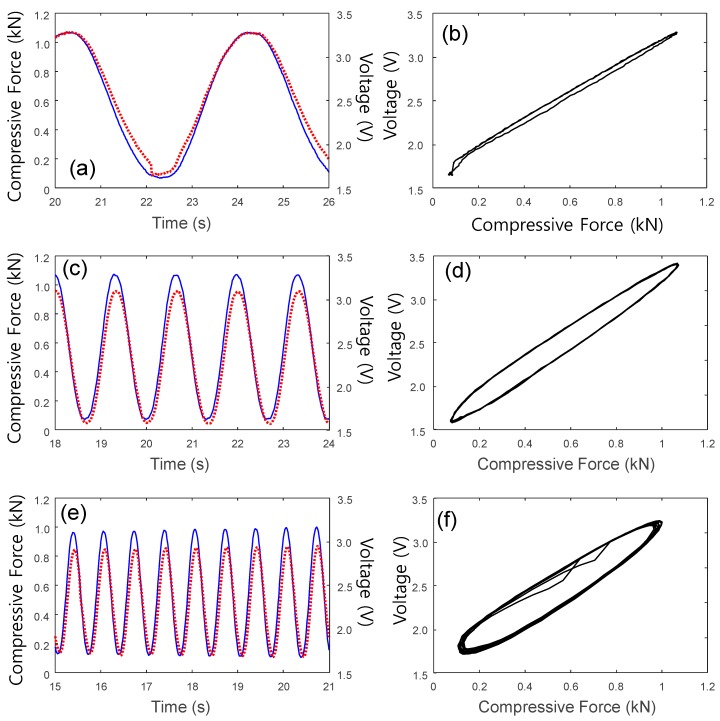
Frequency-dependent dynamic piezoresistive responses to a sinusoidal input and their corresponding hysteresis curves. (**a**,**b**): 0.25 Hz; (**c**,**d**): 0.75 Hz; (**e**,**f**): 1.5 Hz.

In addition, the steady-state piezoresistive response of the CNT/PDMS composite was investigated using the experimental setup shown in Figure 6. As shown in Figure 8, when a sinusoidal compressive load (F≅500±500(2πf⋅t) kN) was applied, the waveform of the measured voltage corresponded well to the compressive load. Generally, the slight phase shift observed between the input loading and voltage output can be characterized using the x-axis to display the applied compressive force, which is called the hysteresis loop between the force input and the voltage output. Because this hysteresis increased slightly when the excitation frequency increased, the nonlinearity of the frequency-dependent hysteresis became obvious as the frequency increased up to 1.5 Hz. Because of this hysteresis behavior, the instantaneous relationship between the force input and voltage output is highly nonlinear. Therefore, estimating the hysteresis model to compensate for this unwanted hysteresis phenomenon is necessary.

## 4. Frequency-Dependent Hysteresis Model and Compensation

Because the hysteresis pattern of the CNT/PDMS composite is similar to those of magnetic and piezoelectric hysteresis, a semilinear Duhem model was considered to capture the loading rate-dependent (or frequency-dependent) hysteresis behavior between the force input and voltage output. This hysteresis model has been demonstrated to be an effective model for magnetic and piezoelectric hysteresis [19]. The equation of the Duhem model under consideration is a first-order nonlinear differential equation. The model for the hysteresis phenomenon between the force input F(t) and the voltage output V(t) can be formulated by:

(5) $$\dot{V}=\alpha \left|\dot{F}\right|\left(f(F)-V\right)+\dot{F}g(F)$$

where f(F) and g(F) are functions that determine the shape of hysteresis loop, and can be chosen as [19]

**Figure 9:**
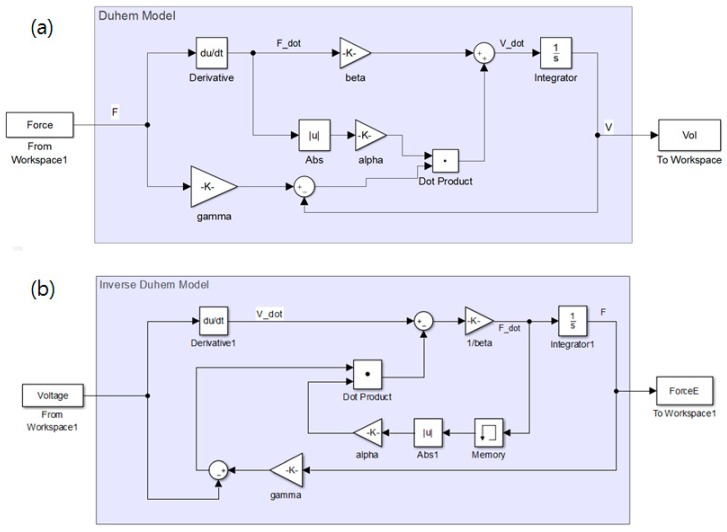
Simulink model. (**a**) semi-linear Duhem hysteresis model and (**b**) inverse model to compensate for the hysteresis.

(6) f(F)=γ⋅F, g(F)=β

where γ and β are constants. Substituting these into Equation (5) yields

(7) $$\dot{V}=\alpha \left|\dot{F}\right|\left(\gamma \cdot F-V\right)+\beta \dot{F}$$

where α>0 and γ>β>0.5α, which are model constants that depend on the shape and area of the hysteresis loop. The voltage output V is considered to be the state variable of the differential equation and is dependent on both the applied force input F and the rate of applied force $\dot{F}$. Such a coupled relationship can induce the nonlinear hysteresis. The nonlinear simulation of Equation (7) with a numerical iterative method (e.g., the built-in Runge-Kutta 4th order method in Simulink [20]) can produce the hysteresis effect with the force measurement, as shown in Figure 9a. Then, an inverse Duhem model as a function of the rate of applied force input can be formulated using the following nonlinear differential equation;

(8) $$\dot{F}=\frac{1}{\beta }\left\{\dot{V}-\alpha \left|\dot{F}\right|\cdot \left(\gamma F-V\right)\right\}$$

## 5. Results and Discussion

**Figure 10:**
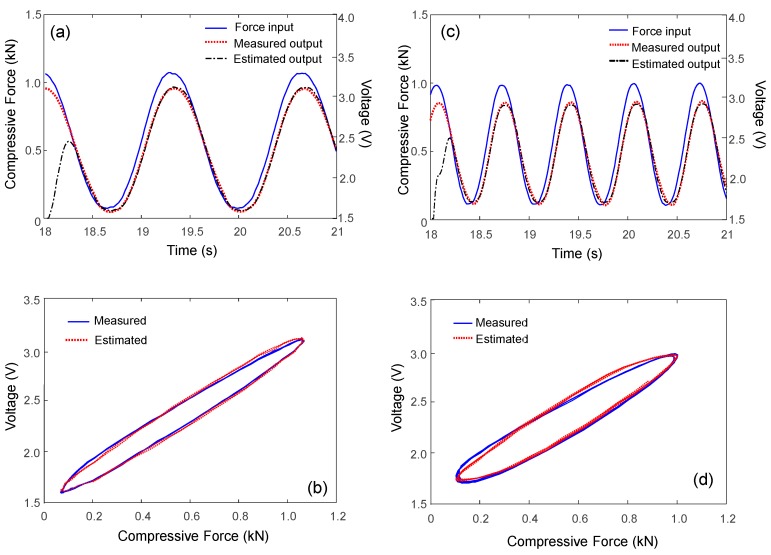
Estimated output based on Duhem hysteresis model. (**a**,**b**) 0.75 Hz; (**c**,**d**) 1.5 Hz.

**Figure 11:**
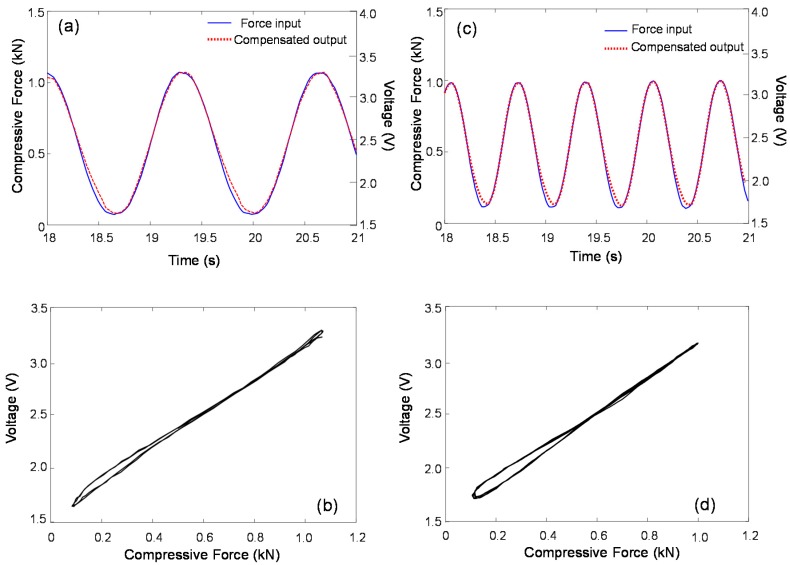
Hysteresis-compensated force responses. (**a**,**b**) 0.75 Hz; (**c**,**d**) 1.5 Hz.

The measured and estimated Duhem model-based hysteresis loops for 1.5 Hz are compared in Figure 10. The Duhem hysteresis model parameter and the two constants are selected to be α = 40, β = 2.4, and γ = 0.9, respectively. The shape of the estimated hysteresis loop corresponded well with that of the measured voltage output. Note that in Figure 10a, the first cycle in the estimated output is transient and not well-matched with the steady-state voltage output because of the use of a zero-initial condition in the Duhem model. This experimental observation implies that the Duhem-model-based estimation can successfully capture the hysteresis with a negligibly small error. The model-based compensated output also agrees well with the force measurement and shows a linear relationship with a slope of unit value, as shown in Figure 11b. This simple hysteresis model can compensate for the nonlinear frequency-dependent hysteresis phenomenon when a dynamic sinusoidal force input is applied.

**Figure 12:**
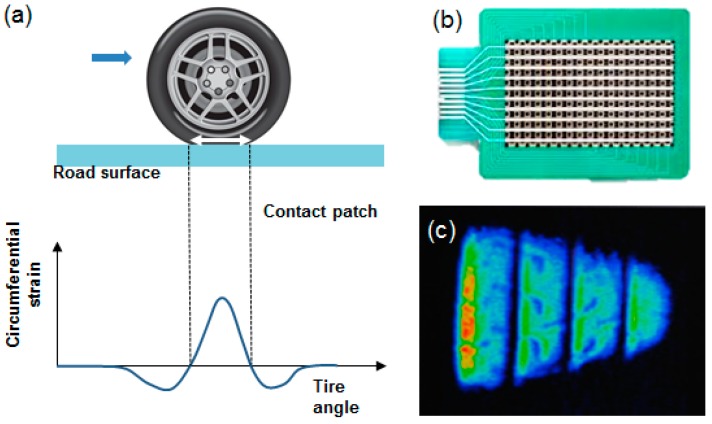
(**a**) Compressive stress distribution on the tire tread, (**b**) matrix array-type force sensor, (**c**) stress (compressive force) distribution on the tire surface.

Although CNT/polymer composites with different polymer matrixes may possess different piezoresistive properties [9], the results of this study at the high compressive force level (approximately 1 kN) demonstrate the possibility of developing a matrix array of force (pressure) sensors for smart automotive tire systems, provided that the new type of CNT composite is based on a synthetic rubber such as a styrene-butadiene rubber (SBR), one of the main components of automotive tires. The tread is the only part of the tire that comes into contact with the road, as illustrated in Figure 12. While a tire is rolling on a hard surface, circumferential strain (deformation) can be induced, as shown in Figure 12a [21]. A matrix array sensor consisting of a few hundred force-sensing element nodes (CNT/polymer-based composite) offers the ability to accurately measure the force (stress) distribution on automotive tire treads caused by large compressive loads (a few kN), as shown in Figure 12b [22].

## 6. Conclusions

In this study, the piezoresistive response of the CNT/PDMS composite was experimentally studied to demonstrate its feasibility as a conductive polymer (i.e., a force-sensitive resistor) for force sensors under a large compressive force (approximately 1 kN). Although the CNT/PDMS composite was fabricated using a simple mixing method, the measured results showed that the electrical resistance of the composite changed in response to a dynamic load as well as different static compressive load levels. Although the CNT/PDMS composite exhibits nonlinear frequency-dependent hysteresis when a dynamic sinusoidal force input is applied, a simple hysteresis model based on the Duhem model can compensate for the frequency-dependent hysteresis.

In the future, we plan to extend this preliminary study in several directions. We will explore more advanced CNT composites based on a synthetic rubber such as a styrene–butadiene rubber (SBR), one of the main components of automotive tires. We also plan to attempt to improve the sensitivity through higher CNT doping levels. Moreover, we will study the application of these composites to in situ tire force-distribution monitoring systems, a promising smart tire sensor technology that has recently emerged in automotive engineering.
